# Artificial intelligence dependency in college students: a critical narrative review

**DOI:** 10.3389/fpsyg.2026.1808268

**Published:** 2026-07-30

**Authors:** Xuehua He, Shan Li, Rongping Cha, Li Wang, Yunxia Shen, Ruijuan Wu, Lili Wu, Xiaoyan Gong, Jingbang Liu

**Affiliations:** Nursing Department, Sir Run Run Shaw Hospital, Zhejiang University School of Medicine, Hangzhou, Zhejiang, China

**Keywords:** artificial intelligence, ChatGPT, college students, dependency, higher education, large language model

## Abstract

The rapid diffusion of generative artificial intelligence (GenAI), particularly large language model (LLM) applications, has fundamentally reshaped university students’ learning, problem-solving, and socio-emotional practices. Alongside clear benefits in efficiency and personalized support, concerns are emerging about AI dependency, understood here as an emerging, non-diagnostic educational-psychological construct rather than a formal clinical disorder. This critical narrative review synthesizes evidence from the past decade on AI dependency among college students, focusing on conceptualizations, theoretical frameworks, prevalence and demographic variations, measurement tools, determinants, intervention strategies, and research gaps. Across studies, an “ask-AI-first” pattern appears increasingly common, although frequency of use should not be equated with maladaptive dependency. The central concern is not AI use itself but patterns of cognitive delegation, emotional reliance, and reduced self-regulation that may be associated with weaker independent engagement, academic-integrity risks, and distress when AI is unavailable. The literature also reveals substantial fragmentation in assessment, although several emerging tools demonstrate promising psychometric properties. Determinants appear multi-level, involving individual vulnerabilities, academic pressure, contextual norms, and technological affordances. Current recommendations emphasize promoting augmentation over automation, strengthening AI literacy and critical evaluation skills, redesigning assessments to preserve cognitive engagement, and implementing supportive interventions for at-risk students. Future research should prioritize longitudinal, cross-cultural, and multi-method designs to clarify developmental trajectories, thresholds for maladaptation, and evidence-based intervention pathways.

## 1 Introduction

In recent years, the rapid advancement of artificial intelligence (AI), particularly generative artificial intelligence (GenAI) and large language model (LLM) technologies such as ChatGPT and DeepSeek, has rapidly transformed modes of human work, daily life, and learning, while also promoting the intelligent development of higher education (OpenAI, 2023; Gibney, 2025; Rouba et al., 2025). Recent surveys have found that the usage rate of generative AI among Chinese college students has reached 99.2%, with 65.9% of students subconsciously prioritizing seeking assistance from AI when encountering problems and 47.1% reporting varying degrees of self-perceived reliance on GAI, with applications covering multiple scenarios ranging from academic research to emotional companionship (Ma et al., 2026; Feng and Zhou, 2025). For master’s students, AI can support the processing of large-scale data and complex research problems, potentially shortening research cycles and facilitating topic formulation, literature retrieval, extraction of key content from readings, research design conceptualization, data collection and processing, and the writing and revision of research outputs (Ren et al., 2025). AI is rapidly becoming an important tool in college students’ learning and research work, profoundly influencing their learning behaviors and cognitive habits (Chen et al., 2025).

Although AI technology can enhance efficiency, provide personalized tutoring, and support creative processes, concerns about AI dependency have emerged as an important psychological and pedagogical issue. In this review, AI dependency is treated as an emerging, non-diagnostic educational-psychological construct, rather than a formal clinical disorder or a synonym for frequent AI use. AI dependency is distinct from mere technology use; it refers to a stable psychological and behavioral tendency to rely disproportionately on AI systems for cognitive processing, academic functioning, affect regulation, or behavioral self-control, particularly when such reliance reduces independent engagement, critical monitoring, or self-regulation (Yang et al., 2025; Wu et al., 2026; Ciudad-Fernández et al., 2025). Yang et al. (2025) distinguished between tool dependency and cognitive dependency and found that cognitive dependency was more strongly associated with cognitive inertia than tool dependency; cognitive inertia was subsequently negatively associated with innovation capability. These findings suggest that the core concern is not AI use itself, but unregulated or excessive psychological reliance that may compromise students’ autonomous cognitive processes under certain conditions. Recent education-focused analyses also caution that unstructured GenAI use may encourage task outsourcing rather than learning-oriented engagement when students lack adequate guidance and metacognitive monitoring (OECD, 2026).

AI dependency appears particularly concerning when examining behavioral patterns in academic contexts. Klimova et al. (2025) surveyed 252 undergraduate students regarding ChatGPT use, finding that 54.4% primarily used AI for augmentation rather than automation. However, students also reported significant concerns about overreliance and diminished critical thinking capabilities. This distinction between augmentation and automation is important because AI-supported explanation, feedback, and idea generation may preserve learning engagement, whereas excessive delegation of writing, reasoning, or decision-making may reduce opportunities for independent practice. Furthermore, AI dependency extends beyond academic tasks into social and emotional domains. Research by Fang et al. (2025) indicated that approximately 60% of college students utilize AI for addressing real-world social scenarios, with specific applications including communication skills training, social strategy planning, and alleviation of social interaction anxiety. With increasing frequency of AI interaction, users’ loneliness and emotional dependency tendencies may increase, and some users may experience distress or a persistent inclination to seek AI companionship when AI support is unavailable. Kirk et al. (2025) pointed out that this socio-emotional dimension of AI dependency reflects a transformation in human-AI relationships. AI has evolved from a purely transactional tool to become integrated into users’ emotional ecology, being perceived by some users as a relationship partner with emotional responsiveness. Recent conceptual work further describes this process as techno-emotional projection, whereby emotionally vulnerable users may project relational needs onto responsive but non-conscious technologies, potentially reinforcing synthetic attachment through repeated interaction (Saracini et al., 2025). When college students actively seek emotional support from AI during periods of emotional distress and view AI as a substitute for interpersonal relationships, this reflects a socio-psychological phenomenon requiring new theoretical perspectives for interpretation (Kirk et al., 2025).

Demographic and contextual variation should also be considered when examining AI dependency among college students. Reported differences by gender, academic level, discipline, socioeconomic status, and urban–rural background may reflect unequal AI access, differences in AI anxiety and AI literacy, disciplinary task demands, and divergent norms regarding acceptable AI use (Chen et al., 2025; Contractor and Reyes, 2025; Singer-Freeman et al., 2025). These variations are relevant because AI use, AI reliance, and AI dependency are not equivalent constructs; the same frequency of use may indicate productive academic support in one context but maladaptive cognitive or emotional overreliance in another. Ethical AI use and academic integrity constitute another important dimension of AI dependency in higher education. When cognitive delegation shifts problem formulation, argument development, authorship, source selection, or revision judgment from students to AI systems, the boundary between assistance and substitution becomes unclear. This may increase risks of undisclosed AI use, AI-assisted ghostwriting, fabricated references, plagiarism, and reduced evidence of individual learning (Cotton et al., 2024; Perkins et al., 2024). However, these risks do not imply that all AI use is misconduct. Rather, they highlight the need to distinguish ethically acceptable augmentation from inappropriate automation and to provide clear guidance on AI-use disclosure, assessment design, and student responsibility for final work.

Unlike well-established constructs such as internet addiction (Young, 1998) or smartphone addiction (Kwon et al., 2013), AI dependency lacks a unified, standardized measurement tool. Morales-García et al. (2024) developed and validated a scale for dependence on artificial intelligence in university students. Recent measurement tools including CAIDS (Chen et al., 2025), LLM-DS (Li et al., 2025), and AIDep-22 (Wu et al., 2026) have been developed to assess this construct systematically. However, these tools differ in target population, AI system type, dimensional structure, and severity threshold; no consensus currently exists on which instrument best captures the construct across educational contexts. At present, these instruments should be understood as research tools for assessing dependency-related tendencies, not as clinical diagnostic measures. This fragmentation has cascading implications: inconsistent findings across studies limit robust meta-analytic synthesis and cumulative knowledge building; the inability to establish normative thresholds hampers progress; and practitioners face uncertainty in selecting appropriate assessment tools.

This critical narrative review addresses critical gaps in understanding AI dependency among college students. Specifically, this review aims to: (1) discuss conceptualizations and theoretical frameworks; (2) synthesize evidence on AI use, reliance, and dependency-related patterns, including demographic and contextual variations; (3) critically evaluate measurement tools; (4) identify determinants contributing to dependency formation; (5) examine intervention strategies; and (6) identify research gaps and future directions. The scope encompasses empirical, psychometric, conceptual, and policy-relevant literature published during the past decade, with a primary focus on college students in higher education settings. Given the conceptual and methodological heterogeneity of this field, the review does not aim to provide pooled effect estimates or diagnostic conclusions. Instead, it seeks to clarify conceptual boundaries, synthesize plausible mechanisms, compare assessment approaches, and identify evidence-informed educational and institutional response strategies.

## 2 Methods

This article was conducted as a critical narrative review. This approach was selected because AI dependency among college students is an emerging and conceptually heterogeneous topic, with evidence distributed across educational psychology, human-AI interaction, psychometrics, digital mental health, academic integrity, and technology-use research. Narrative reviews are suitable for integrating diverse evidence and providing context-specific interpretation when a field is not yet sufficiently mature for pooled quantitative synthesis (Sukhera, 2022). Reporting was informed by SANRA principles, particularly transparent scope definition, search description, scientific reasoning, and appropriate referencing (Baethge et al., 2019).

Search strategy and selection process. Relevant literature was identified through targeted searches of Web of Science (*n* = 187), Scopus (*n* = 156), PubMed/MEDLINE (*n* = 134), PsycINFO (*n* = 98), ERIC (*n* = 76), Google Scholar (*n* = 124), CNKI (*n* = 52), and Wanfang (*n* = 20) from January 1, 2016 to June 1, 2026, yielding an initial identification of 847 records. An additional 23 records were identified through supplementary sources, including reference list screening of relevant articles and expert recommendations, resulting in a total of 870 records. After removing 312 duplicate records identified across databases, 558 unique records remained for title and abstract screening. Following application of the predefined inclusion and exclusion criteria at the title/abstract level, 156 full-text sources were assessed for eligibility. After full-text eligibility review and evidence-relevance appraisal, 64 sources met the inclusion criteria and were retained and cited in the final critical narrative synthesis. Search terms combined three domains: AI-related terms (artificial intelligence, generative AI, large language model, LLM, ChatGPT, chatbot, conversational AI), dependency-related terms (dependency, reliance, overreliance, problematic use, addiction, cognitive offloading, cognitive delegation, automation bias, emotional attachment), and higher-education terms (college students, university students, undergraduates, postgraduates, higher education). Reference lists of relevant articles were also screened. Sources were included when they addressed AI dependency, AI reliance, problematic AI use, AI-related cognitive offloading, emotional attachment to AI, measurement instruments, determinants, outcomes, academic integrity, AI literacy, or intervention strategies in college or higher-education contexts. Studies focused only on general internet or smartphone addiction, purely technical AI development, or non-student populations without transferable relevance were excluded.

Data were synthesized through directed thematic content analysis, which allows predefined themes to guide coding while permitting additional subthemes to emerge from the literature (Hsieh and Shannon, 2005; Braun and Clarke, 2006). Initial themes were defined according to the review aims: conceptualization and theoretical frameworks, prevalence and demographic variation, measurement tools, determinants, possible consequences, ethical and academic-integrity issues, intervention strategies, and future research gaps. Evidence from college student samples was treated as direct evidence, whereas findings from adjacent domains such as general human-AI relationships, digital mental health, AI ethics, and cognitive offloading research were used cautiously to support theoretical interpretation and risk analysis. Because the included sources varied substantially in constructs, instruments, populations, and designs, no meta-analysis or formal risk-of-bias assessment was conducted; instead, stronger interpretive weight was given to longitudinal, experimental, multi-method, and psychometrically validated studies.

## 3 Conceptual frameworks of AI dependency

### 3.1 Defining AI dependency: controversies and challenges

Defining AI dependency remains a contested area in emerging literature. Wu et al. (2026) defined it as excessive academic reliance on AI, whereby emotional regulation, instrumental functioning, higher-order cognition, and behavioral control become increasingly contingent on AI systems. Yang et al. (2025) similarly described AI dependency as a stable tendency to use intelligent systems as continuing cognitive and functional support. In this review, AI dependency is treated as an emerging, non-diagnostic educational-psychological construct rather than a standardized clinical disorder. It refers to disproportionate or poorly regulated reliance on AI systems for academic, cognitive, emotional, or functional support, in which students’ learning agency and self-regulation become increasingly contingent on AI. Adaptive AI use involves purposeful, transparent, and critically monitored assistance. Instrumental reliance refers to routine support for efficiency, such as language polishing, summarization, coding, or feedback. Dependency becomes a concern when reliance is rigid, difficult to inhibit, or associated with distress, reduced autonomous task initiation, diminished critical checking, or functional impairment. Problematic or addiction-like AI use should be reserved for more severe patterns involving loss of control, withdrawal-like discomfort, and persistent negative consequences. Thus, behavioral addiction terminology can be used heuristically, not diagnostically (Ciudad-Fernández et al., 2025; Maral et al., 2025).

### 3.2 Distinguishing AI dependency from related concepts

AI dependency differs from traditional technology addictions through its unique interactive characteristics. Unlike digital tools that mainly deliver content or mediate human-to-human communication, generative and conversational AI systems can produce answers, simulate empathy, personalize feedback, and participate in co-creation (Zhang et al., 2025; Wu et al., 2026). Nevertheless, AI dependency overlaps with adjacent constructs and should be separated from them. General overuse denotes high frequency or duration, whereas dependency involves reduced autonomy, impaired self-regulation, or perceived inability to function without AI. Tool dependency refers to reliance on practical assistance, while cognitive dependency involves outsourcing reasoning, judgment, interpretation, or decision-making (Yang et al., 2025). Emotional dependency refers to using AI for affect regulation, companionship, reassurance, or social rehearsal. Automation bias and trust in automation help explain why students may accept AI outputs without sufficient verification (Parasuraman and Riley, 1997; Lee and See, 2004). Cognitive offloading is also related but not inherently maladaptive: external tools can support learning, but systematic delegation of core learning processes may reduce opportunities for internal processing and skill development (Gerlich, 2025; Peng and Yeh, 2025). Anthropomorphism further blurs the boundary between tool use and relational attachment, as users may attribute agency, empathy, or understanding to systems that only simulate such qualities (Epley et al., 2007; Saracini et al., 2025). Academic misconduct is related but distinct. Cognitive delegation may increase risks of undisclosed AI authorship, ghostwriting, or fabricated references, but not all AI dependency constitutes misconduct. Conceptual boundaries should therefore be based on the delegated function, degree of self-regulatory control, and consequences for learning, well-being, and integrity.

### 3.3 Multidimensional models of AI dependency

Recent studies propose multidimensional models capturing AI dependency’s complexity. Wu et al. (2026) developed a four-dimensional framework including emotional dependence, functional dependence, cognitive dependence, and loss of control. These dimensions correspond to affect regulation, task execution, higher-order cognitive delegation, and behavioral self-regulation. The LLM-D12 scale proposed an alternative dual-dimensional structure, distinguishing instrumental dependency from relational dependency (Yankouskaya et al., 2025). The Dual-Path Framework similarly suggests two interacting routes: instrumental motivations, such as information seeking and efficiency, may foster cognitive reliance; affective motivations, such as loneliness, anxiety, or companionship needs, may foster emotional attachment (Zhai et al., 2025). Together, these models suggest that AI dependency should be assessed as a profile rather than a single score. Different students may show predominantly cognitive, functional, emotional, or control-related patterns, and each profile may require different educational or psychological responses.

### 3.4 Theoretical foundations and integration

AI dependency draws theoretical foundations from multiple psychological frameworks. Behavioral addiction theory explains addiction-like features such as salience, mood modification, withdrawal-like discomfort, conflict, and relapse (Griffiths, 2005; Grant et al., 2010), but it should not be used to diagnose AI dependency without evidence of impaired control, distress, and functional impairment. The I-PACE model links person-related vulnerabilities, affective responses, cognitive biases, and executive control processes (Brand et al., 2019). In college students, these processes may interact with academic stress, loneliness, low self-efficacy, and performance expectations. Technology Acceptance Model (TAM) explains initial adoption through perceived usefulness and ease of use (Davis, 1989), but not maladaptive reliance. Cognitive offloading theory explains the efficiency appeal of AI assistance; however, claims about cognitive debt should remain cautious because current evidence is still emerging and task-specific. Davis’s cognitive-behavioral model of problematic Internet use and compensatory technology-use perspectives help explain why students may use AI to cope with anxiety, loneliness, or academic pressure (Davis, 2001). Self-regulation theory clarifies the role of goal setting, monitoring, impulse control, and strategy adjustment in preventing reliance from becoming loss of control (Zimmerman, 2002). Finally, anthropomorphism and techno-emotional projection explain how emotionally responsive AI may foster synthetic attachment among vulnerable users (Epley et al., 2007; Saracini et al., 2025). Integrating these theories suggests that academic pressure may drive functional dependency; cognitive offloading, automation bias, and excessive trust may drive cognitive dependency; loneliness and anthropomorphic interpretation may drive emotional dependency; and weak self-regulation may convert adaptive reliance into loss of control. This framework emphasizes that AI dependency is not AI use itself, but a mechanism-based continuum from adaptive augmentation to maladaptive overreliance.

## 4 Status and characteristics of AI dependency among college students

AI use and dependency-related patterns have rapidly expanded among college students globally. More precisely, current studies show rapid expansion of AI use and dependency-related patterns, but they should not be interpreted as definitive prevalence estimates for a clinical disorder. U. S. studies reported widespread AI chatbot usage among university students, with 65% using AI tools and 12% reporting weekly engagement patterns (University of California, 2024). In China, surveys reveal high prevalence of AI tool usage, with significant portions classified as heavy users (Zhang et al., 2025). Another study shows substantial increases in generative AI use for academic assignments (Shata and Hartley, 2025). Contractor and Reyes (2025) reported that 80% of students utilize generative artificial intelligence. A Turkish study found 82.5% of students use AI for academic purposes, with significant variance across departments (Kılıç and Koca, 2025). These findings indicate normalization of AI reliance in higher education rather than uniform pathological dependence.

### 4.1 Behavioral and cognitive characteristics

Students primarily use AI for content generation and personal life assistance, followed by academic support, travel planning, and emotional companionship (Shata and Hartley, 2025; Contractor and Reyes, 2025). A defining behavioral characteristic is the “ask AI first” phenomenon where students unconsciously turn to AI when encountering problems (Shata and Hartley, 2025). This pattern may support learning when AI is used for feedback or explanation, but becomes concerning when it replaces initial problem formulation, reasoning, or verification. Research indicates students worry that excessive reliance produces thinking inertia or mental laziness (Zhang et al., 2024). A study of 208 university students found AI dependence significantly correlated with lower self-esteem and increased mind-wandering (Shalu et al., 2025). Preliminary experimental work on AI-assisted writing also suggests possible links between heavy cognitive offloading, weaker recall, and reduced independent engagement, but such evidence remains early and should not be treated as definitive proof of impairment (Kosmyna et al., 2025; Jose et al., 2025).

### 4.2 Demographic and contextual patterns

Usage patterns vary significantly by task type and context. Students differentiate between augmentation, where AI is used to explain concepts, search information, and provide feedback, and automation, where AI directly executes writing, generates images, or produces summaries (Contractor and Reyes, 2025). Augmentation uses correlate with learning support, while automation uses raise concerns about skill atrophy (Singer-Freeman et al., 2025). Academic pressure strongly predicts AI dependency: students under high time pressure or heavy workloads are significantly more likely to use AI than those with lower pressures (Abbas et al., 2024). Differences by gender, academic year, discipline, socioeconomic status, and urban–rural background may reflect unequal access, AI literacy, academic task demands, and policy norms rather than stable personal vulnerability. Temporal patterns show rapid acceleration in AI adoption, with ChatGPT as the dominant platform (Contractor and Reyes, 2025). Usage frequency predicts dependency, and policy contexts matter when AI use is prohibited or ambiguously regulated (Kılıç and Koca, 2025; Singer-Freeman et al., 2025). Overall, productive assistance and maladaptive reliance remain empirically difficult to separate.

## 5 Measurement tools and assessment methods

### 5.1 Existing assessment approaches

The assessment of AI dependency in college students employs three primary methodological approaches. Self-report questionnaires remain the most prevalent, offering standardized measurement through Likert-type scales ranging from 4 to 7 points to quantify cognitive, emotional, and behavioral dimensions of reliance (Sun and Bi, 2025; Wu et al., 2026). These instruments are useful for large-scale surveys but should be treated as measures of dependency-related tendencies rather than diagnostic tools. Behavioral experiments complement self-report measures by quantifying observable dependent behaviors, such as AI recommendation acceptance rates during problem-solving tasks and actual usage patterns in controlled settings (Hou C. et al., 2025; Hou J. et al., 2025). Multimodal or log-based assessment can integrate interaction logs, task performance, and behavioral metrics to capture implicit dependency patterns. Future assessment should combine self-report, task performance, AI-use logs, and verification behavior.

### 5.2 Adapted and newly developed scales

Early AI dependency measurement tools frequently adapted frameworks from established technology addiction scales. The Problematic ChatGPT Use Scale (Yu et al., 2024) modified items from smartphone addiction instruments to assess compulsive AI use patterns, incorporating salience, tolerance, withdrawal, and conflict. Similarly, the Conversational AI Dependence Scale (CAIDS) (Chen et al., 2025) drew from problematic internet use theories, operationalizing withdrawal symptoms, mood modification, and interference with daily functioning. The Artificial Intelligence Dependence Scale (Morales-García et al., 2024) represents a 5-item unidimensional adaptation of general technology addiction measures. These adapted scales provide early evidence but may insufficiently capture AI-specific features such as cognitive offloading, prompt-based co-creation, anthropomorphic interaction, and overtrust in fluent outputs.

Recent years have witnessed the emergence of specialized scales explicitly designed to assess AI dependency. The AI Dependence Scale (AIDep-22) (Wu et al., 2026) operationalizes four dimensions: emotional dependence, functional dependence, cognitive dependence, and loss of control. The AI Dependence Questionnaire (AID-Q) (Sun and Bi, 2025) offers a concise 12-item instrument distinguishing functional and emotional reliance. For large language models, LLM-D12 (Yankouskaya et al., 2025) measures instrumental and relational dependency. The Clinical AI Dependency Assessment Scale (CAIDAS) (The AI Addiction Center, 2025) proposes a broader clinical profile, whereas the Scale of Reliance Behaviors (Hou C. et al., 2025; Hou J. et al., 2025) focuses on problem-solving reliance. Because some instruments are new, preprint-based, or developed outside student samples, independent validation remains necessary.

### 5.3 Psychometric properties and methodological issues

Established AI dependency scales demonstrate robust psychometric properties across multiple validation studies. Internal consistency reliability is consistently high, with Cronbach’s alpha coefficients ranging from 0.87 to 0.92 for widely used instruments like AIDep-22, AID-Q, and CAIDS (Sun and Bi, 2025; Wu et al., 2026). Test–retest reliability shows good stability, and confirmatory factor analyses support proposed multidimensional structures (Chen et al., 2025; Wu et al., 2026). Nevertheless, psychometric promise should not be confused with construct consensus. Current tools measure overlapping but non-identical targets, including general AI dependence, problematic ChatGPT use, conversational AI dependence, academic AI dependence, and LLM-specific instrumental or relational dependency. Convergent validity is commonly examined through correlations with AI trust and general technology use, whereas criterion-related validity should be further tested against academic self-efficacy, critical thinking, subjective well-being, and objective learning outcomes. More evidence is needed on predictive validity, measurement invariance, sensitivity to change, and objective educational outcomes.

Current measurement approaches face several critical methodological challenges. First, over-reliance on self-report measures introduces common method bias and social desirability effects. Behavioral experiments, while more objective, often suffer from limited ecological validity. Second, cross-cultural validation is limited, as most scales have been developed in Western or East Asian contexts (Yankouskaya et al., 2025). Third, construct validity concerns persist due to blurred boundaries between AI dependency, AI trust, and general attitudes toward technology (Lee and See, 2004). Fourth, longitudinal studies tracking dependency trajectories remain scarce (Wu et al., 2026). Finally, the rapid evolution of AI technologies risks rendering static measurement instruments obsolete. At this stage, no instrument should be treated as definitive; existing scales are best used to identify patterns, dimensions, and risk profiles requiring further validation. A practical measurement priority is to assess purpose, task context, degree of cognitive delegation, emotional reliance, loss of control, verification behavior, and functional consequences rather than usage frequency alone. See comparison of AI dependency-related measurement instruments in Table 1.

**Table 1:** Comparison of AI dependency-related measurement instruments.

Instrument	Target construct	Population/context	Main dimensions or focus	Strengths	Limitations/cautions
Dependence on Artificial Intelligence Scale/DAI (Morales-García et al., 2024)	General AI dependence	University students	Usually treated as a brief/unidimensional AI dependence measure	Efficient screening tool; early validation in university population	Limited dimensional coverage; cannot fully distinguish instrumental use from maladaptive dependency
Problematic ChatGPT Use Scale/PCUS or PCGUS (Yu et al., 2024; Maral et al., 2025)	Problematic or compulsive ChatGPT use	ChatGPT users/student samples	Addiction-like features such as salience, mood modification, withdrawal-like discomfort, conflict, and loss of control	Specific to ChatGPT; useful for identifying problematic-use tendencies	Risk of over-pathologizing frequent academic use; requires evidence of impairment for stronger claims
Conversational AI Dependence Scale/CAIDS (Chen et al., 2025)	Dependence on conversational AI systems	Chinese college students	Uncontrollability, withdrawal symptoms, mood modification, and negative impact	Captures chatbot-specific dependence and affective/behavioral features	Further cross-cultural validation is needed; overlap with AI trust and engagement should be examined
AIDep-22 (Wu et al., 2026)	Academic AI dependency	Chinese undergraduates	Emotional dependence, functional dependence, cognitive dependence, and loss of control	Multidimensional and closely aligned with higher-education contexts	Longitudinal predictive validity, cross-cultural invariance, and behavioral validation remain needed
LLM-D12/LLM-DS (Li et al., 2025; Yankouskaya et al., 2025)	Large language model dependency	LLM users/student-relevant contexts	Instrumental dependency and relational/relationship dependency	Distinguishes task-oriented reliance from relational attachment	Newly developed; student-specific norms and cross-cultural validation are still limited
Scale of Reliance Behaviors during problem solving (Hou C. et al., 2025; Hou J. et al., 2025)	Reliance behavior during AI-supported problem solving	Undergraduate learning/problem-solving tasks	Reflective, cautious, reckless, and collaborative reliance patterns	Assesses task behavior rather than only symptoms; useful for educational settings	Measures reliance strategies more than dependency severity; ecological validity depends on task design
SAID and academic-task AI dependence tools (Garcia Castro et al., 2025)	AI dependence in academic tasks	Academic/educational contexts	Task delegation, trust, informative exclusivity, and academic reliance indicators	Strong relevance to coursework and assessment contexts	Dimensional structures vary; integration with broader dependency frameworks is needed

The table distinguishes assessment instruments by construct coverage rather than treating all scales as equivalent diagnostic tools. Verify final bibliographic details before submission, especially for recently published or preprint sources.

## 6 Factors of AI dependency

### 6.1 Individual-level factors

Individual psychological and demographic variables significantly predict AI dependency levels among college students. Because most evidence is cross-sectional, these variables should be interpreted as correlates or risk indicators rather than confirmed causes. Male students show significantly higher overall AI dependency levels than females, with males more likely to use AI for automation tasks and females for augmentation (Chen et al., 2025; Singer-Freeman et al., 2025). Women also report lower perceived AI knowledge and higher AI-related anxiety (Russo et al., 2025). Academic year shows variation: first-year students are often more conservative and anxious, while senior and graduate students view AI as beneficial for complex tasks (Singer-Freeman et al., 2025). Departmental differences are pronounced, with natural science majors reporting higher AI usage than humanities majors (Contractor and Reyes, 2025; Kaya et al., 2024). Socioeconomic and geographic differences may reflect access, task demands, and AI literacy (Chen et al., 2025).

AI self-efficacy demonstrates robust positive correlations with dependency. Individuals scoring higher on AI-Self Efficacy Scale dimensions show significant positive correlations with higher dependence. However, general self-efficacy shows no significant correlation with AI dependence, contradicting some prior research linking academic self-efficacy to dependency (Zhang et al., 2024). Thus, confidence in using AI may increase exposure and reliance, whereas academic self-efficacy may protect against substituting AI for personal competence. Longitudinal studies reveal critical temporal predictors. Baseline loneliness, emotional dependence, and problematic usage predict corresponding states after 4 weeks of chatbot interaction (Fang et al., 2025). Anxiety predicts escapism and social motivations for AI use, while depression predicts escapism motivation (Hou C. et al., 2025; Hou J. et al., 2025). Academic pressure remains especially important: weekly usage exceeding 4 hours and academic stress levels show high impact on AI dependency scores (Hough et al., 2025; Zhang, 2025). Academic stress, low perceived competence, anxiety, loneliness, and desire for immediate relief may therefore interact to encourage instrumental and emotional reliance.

### 6.2 Technological and design factors

Technological characteristics of AI systems substantially influence dependency formation. Perceived anthropomorphism emerges as a significant positive predictor, suggesting that the more human-like users perceive AI to be, the more they rely upon it (Chen et al., 2025). Conversational fluency, empathy simulation, personalization, and immediate responsiveness may increase perceived social presence and trust. This finding is consistent with an emerging preprint on the AI Genie phenomenon, which describes how desired outcomes with minimal effort may produce addiction-like tendencies (Shen et al., 2026). The same preprint proposes three dependency-related patterns: Escapist Roleplay, Pseudosocial Companion, and Epistemic Rabbit Hole (Shen et al., 2026). Chatbot design elements may reinforce repeated use through non-deterministic, empathetic, and agreeable responses, along with notifications and immediate visual presentation of outputs (Shen et al., 2026). Emotional and efficiency needs jointly drive AI chatbot dependence, with productivity enhancement perceptions showing stronger effects than emotional needs (Liu, 2025). Thus, low friction, rapid reward, and anthropomorphic cues may lower the threshold for repeated reliance.

### 6.3 Environmental and social factors

Environmental and contextual factors create conditions fostering dependency. Academic workload and time constraints function as primary drivers; over 80% of educators report heavy institutional workload as key to AI dependence (Alhur et al., 2025). Students under high time pressure or heavy workloads are 2.3 times more likely to use AI for assignments (Singer-Freeman et al., 2025). Use intensity may be associated with dependency-related scores, but frequency alone is insufficient to define maladaptive dependence (Kılıç and Koca, 2025; Wu et al., 2026). Social exclusion indirectly impacts problematic use through loneliness, fear of negative evaluation, and social anxiety as mediators (Shen et al., 2026). Ambiguous AI policies, inconsistent faculty expectations, automatable assignments, and limited academic support may further encourage hidden or excessive use, whereas clear rules, tutoring, AI literacy, and human support may moderate dependency risk.

## 7 Discussion

### 7.1 Synthesis of key findings

This critical narrative review synthesizes evidence on AI dependency among college students, revealing a complex phenomenon characterized by high AI use rates, dependency-related patterns, multidimensional manifestations, and determinants that distinguish it from traditional technology addictions. The revised synthesis treats AI dependency as an emerging, non-diagnostic construct involving maladaptive or disproportionate reliance rather than as a confirmed clinical addiction. These findings reveal a fundamental shift in how students engage with technology. Generative AI can function as tutor, writing partner, search assistant, decision aid, and emotional companion, making dependency more complex than screen time or passive consumption. The phenomenon of cognitive debt suggests that AI dependency’s core mechanism involves systematic displacement of internal processing rather than simply excessive screen time. However, cognitive debt remains an emerging concern rather than a settled causal conclusion.

The review identifies a critical fragmentation in measurement approaches that hinders field advancement. While recent instruments like AIDep-22 (Wu et al., 2026), CAIDS (Chen et al., 2025), and LLM-D12 (Yankouskaya et al., 2025) demonstrate robust psychometric properties, the lack of consensus on operationalizing the construct precludes meta-analysis and normative data establishment. The central theoretical tension is whether AI dependency should be conceptualized as addiction-like use or as technology integration that becomes problematic only when autonomy, control, or functioning is impaired. Ciudad-Fernández et al. (2025) caution against premature pathologization; therefore, this review distinguishes adaptive AI use, instrumental reliance, dependency-related overreliance, and problematic or addiction-like use.

### 7.2 Evidence strength and ethical implications

The determinants analysis reveals nuanced patterns that challenge simplistic dependency narratives. Contrary to assumptions that AI dependency primarily reflects moral failure or academic dishonesty, findings indicate it emerges from complex interactions of individual psychology, technological affordances, and environmental pressures. Academic workload and time constraints function as primary drivers (Singer-Freeman et al., 2025). The socio-emotional dimension identified by Kirk et al. (2025) also suggests roots in unmet psychological needs. Most evidence remains cross-sectional, exploratory, psychometric, or short-term observational. This review therefore does not claim that AI dependency directly causes cognitive impairment, academic decline, or emotional dysfunction; longitudinal, experimental, and digital-trace studies are needed to clarify directionality.

This review contributes several insights to the literature. First, evidence from diverse geographical contexts suggests that AI dependency is a global phenomenon requiring cross-cultural validation. Second, the multidimensional framework distinguishing cognitive from tool dependency (Yang et al., 2025) explains why some students benefit from AI assistance while others develop maladaptive reliance. Third, baseline loneliness and emotional dependence may offer prevention points (Fang et al., 2025). Finally, AI dependency raises academic-integrity concerns when cognitive delegation transfers authorship, reasoning, source selection, or problem formulation to AI without disclosure. Policies should distinguish ethical augmentation from inappropriate automation (Cotton et al., 2024; Perkins et al., 2024).

### 7.3 Intervention and prevention strategies

The implications for higher education practice are substantial. Rather than framing AI use in binary prohibited/allowed terms, institutions should foster “augmentation over automation” approaches that preserve cognitive engagement while leveraging AI’s efficiency benefits (Contractor and Reyes, 2025). Training programs should explicitly address the distinction between using AI for productivity enhancement versus cognitive outsourcing, targeting the self-regulation deficits that characterize dependency rather than restricting technology access. Intervention should be mechanism-oriented because academic stress, low self-efficacy, cognitive delegation, emotional attachment, and loss of control require different responses.

#### 7.3.1 Cognitive behavioral and self-regulation interventions

Cognitive behavioral approaches offer promising frameworks for addressing problematic or severe dependency-related AI use among college students. Davis (2001) cognitive-behavioral model of pathological Internet use provides a foundation, emphasizing maladaptive cognitions, psychosocial problems, and behavioral reinforcement as key mechanisms. In the AI context, “pathological” should be used cautiously; CBT-informed strategies are most appropriate for students showing loss of control, distress when AI is unavailable, or functional impairment. Adapted for AI contexts, CBT interventions target cognitive distortions such as overestimation of AI capabilities, catastrophizing about inability to function without AI, and beliefs that AI substitutes for human relationships. Behavioral components include self-monitoring, planned AI-free periods, and skill-building exercises for tasks previously delegated to AI. Grant et al. (2010) and Griffiths (2005) inform interventions targeting salience, mood modification, tolerance, and withdrawal-like discomfort. For students driven by academic stress or low self-efficacy, universities should provide writing centers, tutoring, time-management training, and staged assignments that rebuild mastery rather than merely restrict AI access.

#### 7.3.2 AI literacy education and academic integrity

Comprehensive AI literacy programs represent preventive approaches equipping students with critical thinking skills and balanced perspectives on AI capabilities. Rather than prohibition, educational interventions focus on developing AI competency that includes understanding technological limitations, recognizing biases, and maintaining human agency in decision-making. OECD (2026) emphasizes that effective AI literacy education must move beyond technical skills to include ethical reasoning, critical evaluation of AI outputs, and understanding societal implications. Consistent with international guidance, AI literacy should also include disclosure norms, data privacy, authorship responsibility, and awareness of hallucinated outputs (UNESCO, 2023). Contractor and Reyes (2025) found that students receiving structured AI literacy training demonstrated lower dependency scores compared to controls. Curriculum components should include AI limitations, critical evaluation of generated content, academic-integrity expectations, human-AI collaboration, and recognition of dependency warning signs. Specific activities may include “think-first-then-prompt” exercises, AI-free first drafts, hallucination detection, source triangulation, AI-use reflection logs, and assignment-specific disclosure statements. Importantly, AI literacy education must be integrated across disciplines rather than isolated to computer science departments. Assessment policies should distinguish AI-permitted, AI-assisted, AI-restricted, and AI-free tasks, using process portfolios, oral defense, in-class writing, and version histories to preserve evidence of student learning (Perkins et al., 2024).

#### 7.3.3 Emotional dependence and human support

AI dependency extends beyond academic tasks into emotional and relational domains. Students experiencing loneliness, anxiety, social discomfort, or academic failure may use AI for reassurance, companionship, and coping. Interventions should therefore address emotional distress directly rather than treating AI use only as misconduct or poor study behavior. Recommended strategies include emotional AI literacy, psychoeducation on human-AI relational boundaries, peer-support programs, counseling referral pathways, and stronger human support networks. Students should reflect on whether AI interaction supplements or replaces human relationships; those distressed when AI is unavailable may benefit from CBT-informed coping plans and gradual re-engagement with human social support.

#### 7.3.4 Environmental design and policy frameworks

Institutional-level interventions create environments supporting balanced AI use through policy, infrastructure, and cultural norms. Comprehensive AI governance frameworks at universities should establish clear guidelines distinguishing permissible augmentation from problematic automation. Alhur et al. (2025) found that institutions with explicit AI use policies showed lower rates of problematic dependency compared to unregulated environments, though overly restrictive policies correlated with underground usage and increased anxiety. Effective policies include transparent assignment guidelines, clear academic-integrity definitions, AI-free assessment components, first-year AI literacy training, and support systems for students struggling with AI-related anxiety. Infrastructure considerations include monitoring concerning usage patterns only when privacy protections and student consent are clear, while providing accessible alternatives such as tutoring, writing support, and counseling services. Policy should be educative and supportive rather than purely punitive, because fear of punishment may increase concealment and reduce help-seeking.

#### 7.3.5 Effectiveness and implementation challenges

Despite growing intervention efforts, significant challenges limit effectiveness and widespread implementation of AI dependency prevention programs. Research indicates that intervention efficacy varies substantially based on individual differences, with outcomes moderated by gender, academic discipline, personality traits, and cultural background (Zhang et al., 2025). Implementation barriers include student and faculty resistance, resource constraints, rapid AI evolution, lack of standardized monitoring tools, and cultural variability in appropriate AI use norms. Evaluation should include dependency scores, AI-free task performance, critical thinking, academic self-efficacy, stress, loneliness, disclosure compliance, and fairness perceptions. Yang et al. (2025) identified that interventions focusing solely on individual behavior change show limited long-term success without environmental modifications. Additionally, the distinction between productive AI assistance and pathological dependency remains blurry in practice, creating confusion for students, educators, and mental health professionals. Evaluation studies report mixed evidence regarding sustainability, with initial positive effects frequently diminishing without ongoing support. Future studies should test intervention components through longitudinal, experimental, and quasi-experimental designs before strong claims of effectiveness are made. See mechanism-targeted intervention and prevention framework for AI dependency among college students in Table 2.

**Table 2:** Mechanism-targeted intervention and prevention framework for AI dependency among college students.

Mechanism/risk pathway	Risk manifestation	Intervention target	Practical strategies	Evaluation indicators
Academic stress and low academic self-efficacy	Students use AI to avoid difficult tasks, accelerate assignments, or compensate for low confidence	Academic coping and mastery experiences	Writing center, tutoring, statistics/coding support, time-management training, staged assignments, AI-free practice before AI-assisted feedback	Academic self-efficacy, perceived stress, AI-free task completion, help-seeking
Cognitive offloading and cognitive delegation	AI performs reasoning, writing, summarizing, problem formulation, or decision-making for the student	Critical engagement and independent cognitive effort	Think-first-then-prompt exercises, AI-free first drafts, AI-output critique, reflective logs, concept mapping before AI use	Critical thinking tasks, independent writing quality, source verification accuracy, self-explanation quality
Automation bias and excessive trust	Students accept AI outputs, references, or explanations without checking accuracy	Verification behavior and metacognitive monitoring	Hallucination-detection tasks, source triangulation, prompt audits, comparison with authoritative sources, factual-error analysis	Accuracy-checking performance, proportion of verified claims, ability to identify fabricated references
Emotional compensation and AI attachment	AI is used as companionship, reassurance, social rehearsal, or substitute for human support	Emotional regulation and human social connection	Emotional AI literacy, peer-support groups, counseling referral, psychoeducation on human-AI relational boundaries, alternative coping plans	Loneliness, distress, relational AI dependence, help-seeking readiness, perceived human support
Loss of control	Unplanned, compulsive, or difficult-to-limit AI use; distress when AI is unavailable	Self-regulation and behavioral boundaries	Usage diary, digital boundary plan, graded reduction, CBT-informed cognitive restructuring, motivational interviewing	Loss-of-control scores, use frequency by purpose, distress when AI unavailable, functional impairment
Academic integrity ambiguity	Undisclosed AI-generated work, ghostwriting, fabricated citations, or unclear authorship	Transparent authorship and ethical AI use	AI-use disclosure templates, AI-permitted/AI-restricted task categories, oral defense, process portfolio, draft history, version logs	Disclosure compliance, integrity incidents, authorship transparency, student fairness perceptions
Digital divide and unequal AI literacy	Unequal access, uneven prompt skills, and inconsistent understanding of ethical AI use	Equity and inclusive AI competence	Accessible AI literacy modules, discipline-specific guidance, culturally responsive examples, faculty training, alternative supports for low-access students	AI literacy, perceived fairness, digital access, course-level consistency
Anthropomorphic or persuasive design affordances	Students over interpret AI empathy, agency, or authority, increasing emotional or epistemic reliance	Design awareness and critical human-AI relational literacy	Explain simulation vs. understanding, discuss anthropomorphism, teach limits of AI agency, promote human-in-the-loop norms	Anthropomorphism awareness, uncritical trust, relational-dependence indicators

The framework is intended for educational and institutional planning. Intervention effectiveness should be tested through longitudinal, experimental, or quasi-experimental designs.

### 7.4 Limitations and future research

Several limitations warrant acknowledgment. First, the rapid pace of AI technology development creates challenges for capturing the most current trends, with potentially relevant studies published after our search window. Second, significant heterogeneity exists across studies in methodological approaches, population characteristics, and measurement instruments, limiting direct comparability and meta-analytic synthesis. Third, because this article is a critical narrative review rather than a formal systematic review or meta-analysis, selection and interpretation may still be influenced by author judgment despite a transparent search strategy, eligibility criteria, and thematic synthesis. Fourth, most measurement instruments demonstrate cross-cultural validation limitations. Fifth, self-report bias may lead to underreporting of excessive AI use. Sixth, longitudinal studies tracking dependency trajectories remain scarce, hindering understanding of temporal dynamics, causal relationships, and long-term outcomes. Finally, the distinction between productive AI assistance and maladaptive dependency-related use remains blurred in practice. Evidence from adjacent domains was used to inform interpretation but should not be treated as direct proof among college students. These limitations constrain the strength of practical recommendations and require cautious interpretation by educators and policymakers.

Multiple promising directions emerge for advancing understanding of AI dependency. First, longitudinal studies tracking students across academic careers are urgently needed to establish temporal sequences, identify critical transition points, and differentiate adaptive from maladaptive usage patterns. Second, cross-cultural validation research should examine how cultural values, technological infrastructure, and regulatory environments influence AI dependency. Third, multi-method approaches combining self-report, behavioral experiments, physiological data, and digital trace analysis can provide more objective assessment. Fourth, intervention studies are needed to identify effective treatment components and establish evidence-based guidelines. Fifth, neurobiological research should further investigate the cognitive debt phenomenon. Finally, policy implementation research should evaluate real-world effectiveness of different AI governance frameworks. Future work should also examine academic integrity, disclosure practices, emotional attachment, and equity in AI access as core dimensions of dependency-related risk.

## 8 Conclusion

This critical narrative review indicates that AI dependency is emerging as a multifaceted risk in higher education, extending beyond frequent use to include cognitive delegation, emotional reliance, and diminished self-regulation. It should be understood as an emerging, non-diagnostic construct rather than a confirmed clinical addiction. Evidence synthesized here suggests that the central concern is not screen time, but potential cognitive debt, namely systematic offloading of higher-order thinking that may be associated with weaker memory and critical reasoning over time. Because current evidence remains heterogeneous and largely correlational, such consequences should be interpreted as plausible risks requiring further testing. At the same time, progress in the field is constrained by fragmented measurement practices. Practically, universities should promote augmentation over automation, paired with AI literacy, assessment redesign, and supportive identification of at-risk students. Future work should prioritize longitudinal, cross-cultural, and multi-method studies to clarify developmental trajectories and intervention thresholds.
